# Intraoperative electrocorticography in focal drug-resistant epilepsy: A 10-year retrospective single-center study

**DOI:** 10.3892/br.2025.2049

**Published:** 2025-09-01

**Authors:** Amani Alshahrani, Ziyad Mohammed Althani, Raidah Al-Baradie, Tarek Jallul, Faisal Alotaibi, Ahmed Najjar, Mona Faraidy, Brent Hedgcock, Shahid Bashir, Ali Mir

**Affiliations:** 1 Department of Adult Neurology, King Fahad Specialist Hospital, Dammam 31444, Saudi Arabia; 2 Department of Pediatric Neurology, King Fahad Specialist Hospital, Dammam 31444, Saudi Arabia; 3 Department of Neurosurgery, King Fahad Specialist Hospital, Dammam 31444, Saudi Arabia; 4 Neuroscience Centre, King Faisal Specialist Hospital and Research Center, Riyadh 11211, Saudi Arabia; 5 Department of Surgery, College of Medicine, Taibah University, Almadinah Almunawwarah 41411, Saudi Arabia; 6 Department of Anesthesia, King Fahad Specialist Hospital, Dammam 31444, Saudi Arabia; 7 Department of Neurophysiology, King Fahad Specialist Hospital, Dammam 31444, Saudi Arabia; 8 Neuroscience Center, King Fahad Specialist Hospital Dammam, Dammam 31444, Saudi Arabia; 9 King Salman Center for Disability Research, Riyadh 11614, Saudi Arabia

**Keywords:** intraoperative electrocorticography, epilepsy surgery, retrospective study, single center, neurosurgery, brain mapping

## Abstract

Intraoperative electrocorticography (ECoG) represents a crucial tool for improving seizure outcomes during epilepsy surgeries by assisting in localization of the epileptogenic zones. There is a shortage of information in the literature regarding single-center experiences and long-term outcomes after ECoG-guided surgeries. Data are particularly scarce from the Eastern Mediterranean Region. The aim of the present retrospective study was to investigate the effectiveness of ECoG-guided epilepsy surgeries performed in a single center in terms of seizure outcomes. The study included 30 patients with drug-resistant epilepsy who underwent ECoG-guided surgeries between January 2013 and December 2024. Participant details, clinical history and data, surgical interventions, and outcomes were all reviewed retrospectively. The intraoperative findings were assessed and post-resection ECoG was used to define the extent of resections. Of the 30 patients, 19 (63.3%) achieved complete seizure freedom (Engel Class I) post-surgery. ECoG revealed zones of preexisting epileptogenic activity in all patients undergoing the procedure, with 43.3% of patients showing no residual interictal discharges on ECoG after resection. ECoG remains relevant in achieving improved outcomes in epilepsy surgery, with seizure freedom accurately predicted by post-surgical ECoG silence.

## Introduction

Approximately one-third of patients with drug-resistant epilepsy (DRE) suffer from a structural lesion that can be treated surgically (1,2). Identifying these lesions is important, as they often represent the ictal onset zone. Epilepsy surgery targeting these lesions can lead to a significant reduction in seizure frequency and improve quality of life. Patients with lesions identified through magnetic resonance imaging (MRI) were more likely to experience seizure freedom following resection, highlighting the importance of imaging in treatment planning (3). Even when phase I presurgical evaluations yield concordant data, 30-50% of surgical candidates still experience seizure recurrence (4,5). Failure of epilepsy surgery for focal epilepsy may be caused by insufficient excision of the epileptogenic zone (EZ) or by inadequate disruption of pathogenic hubs within the epileptogenic network (6).

An intraoperative electrocorticography (ECoG) method was developed by Penfield and Jasper in the 1930s. This method involved placing electrodes directly on surgically exposed cortical surfaces and recording brain activity before and after resection. This process allowed the identification of the irritative zone as well as the EZ intraoperatively (7). ECoG is particularly advantageous for patients with neocortical lesions that cause temporal or extratemporal epilepsy. This approach has been utilized in patients with DRE stemming from either temporal or extratemporal tumors, mesial temporal sclerosis (MTS) and focal cortical dysplasia (FCD). It can be utilized to prevent the need for long-term extraoperative invasive electroencephalography (EEG) in carefully selected individuals (8,9). However, it cannot replace the presurgical phase I evaluation, as precise lateralization and localization of the ictal onset zone or a strong hypothesis of the EZ is necessary before performing ECoG.

ECoG effectiveness has been well documented in high-resource settings, particularly across academic centers in North America and Europe (9). However, evidence from the Eastern Mediterranean Region (EMR), including countries such as Saudi Arabia, Egypt, Jordan, and Lebanon remains limited. This lack of data is concerning, as healthcare systems in the EMR often face distinct challenges such as restricted access to specialized epilepsy surgery programs, disparities in diagnostic resources, and delayed referral (10). Moreover, existing studies from the region are typically limited to small case series and rarely report long-term seizure outcomes, making it difficult to assess the broader utility and effectiveness of ECoG-guided surgical interventions.

To address this gap, a retrospective analysis of ECoG-guided epilepsy surgeries performed at a tertiary care center in Saudi Arabia was conducted. The objective of the present study was to fill this gap by assessing whether postresection ECoG silence is predictive of Engel Class I outcomes.

## Patients and methods

The present retrospective observational study involved 30 patients with DRE who underwent ECoG-guided epilepsy surgery as part of the comprehensive epilepsy program at King Fahad Specialist Hospital in Dammam, Saudi Arabia, between January 2013 and December 2024. Patients with a minimum follow-up duration of 6 months were recruited. Clinical and demographic information, such as sex, handedness, central nervous system examination, seizure type, epilepsy duration, MRI findings, age at surgery, and follow-up duration, were obtained. Participants with incomplete data were excluded. The present study received ethics approval (approval no. NEU0402) from the Ethics Committee of King Fahad Specialist Hospital (Dammam, Saudi Arabia). Given the retrospective nature of the study and the use of de-identified clinical data, the requirement for patient consent for participation in the present study was waived by the Ethics Committee of King Fahad Specialist Hospital.

All patients underwent phase I presurgical evaluation in the ABRET-accredited Epilepsy Monitoring Unit. The patients were monitored with continuous noninvasive video EEG using a 10-20 electrode placement system with additional left and right anterior temporal T1 and T2 electrodes. On average, at least two seizures were recorded. Preoperative imaging studies included 1.5 Tesla or 3 Tesla MRI scans. Lesions were categorized as temporal, extratemporal, or both temporal and extratemporal. Patients with normal MRI scans underwent advanced modalities, such as positron emission tomography (PET) or single photon emission computed tomography (SPECT) scans to localize or lateralize the EZ. All patients were reviewed during the weekly multidisciplinary epilepsy surgery care conference. Both the patients and their relatives were informed about the need for EcoG, and written informed consent was obtained.

All patients underwent the procedure under general anesthesia. Anesthesia was induced with propofol at a dose of 2-3 mg/kg, fentanyl at 2 mcg/kg, and rocuronium at 0.5 mg/kg. After intubation, arterial and peripheral lines were secured. An infusion of remifentanil at a rate of 0.1-0.3 mcg/kg/min, in combination with inhaled sevoflurane at 1 MAC, was used for anesthetic maintenance. After 2021, due to a nationwide shortage, remifentanil was replaced by dexmedetomidine infusion at a rate of 0.2-0.5 mcg/kg/h. Rocuronium at a dose of 0.2 mg/kg was given as needed, especially before ECoG. During ECoG recordings, the sevoflurane concentration was reduced to 0.2 MAC to decrease anesthesia levels. The dexmedetomidine infusion at 0.2-0.5 mcg/kg/h and rocuronium at 0.2 mg/kg/dose were continued throughout the ECoG recording. Sevoflurane was increased back to 1 MAC at the end of each ECoG recording session. ECoG was performed during surgery to detect the presence of interictal discharges (IEDs) and to assist in making surgical decisions regarding the extent of resection of the EZ. ECoG was conducted both before and after resection to monitor for IEDs in the cortical areas and to help guide the surgeons in achieving maximal or complete excision of the epileptogenic regions. In total, four to six contact strip electrodes were utilized for recording. Engel's classification method was used to determine the outcomes of seizures (Class I-IV), with Class I defined as completely seizure-free (11).

The data were summarized using descriptive statistics, covering variables such as gender, age at surgery, duration and type of seizures, lesion type identified on MRI, surgical approach, pre- and post-resection ECoG findings, histological results, as well as postoperative MRI and EEG evaluations. A postoperative CT scan of the brain was performed on all patients to assess complications such as hemorrhage, infarction, and hydrocephalus. Post-operative MRI was performed only if clinically necessary due to logistical constraints at King Fahad Specialist Hospital (Dammam, Saudi Arabia). Due to the small sample size, descriptive statistics were used to summarize patient demographics and clinical outcomes, including means, medians, and ranges for continuous variables, and frequencies and percentages for categorical variables. All analyses were performed using IBM SPSS Statistics for Windows, Version 26.0 (IBM Corp.). No multivariate analysis was performed due to sample size limitations.

## Results

In the present study, a total of 30 patients were included, consisting of 16 males and 14 females. Of these, 26 patients were right-handed and 4 patients were left-handed. A total of 28 patients had a normal neurological examination, while two had right hemiparesis. In terms of seizure type, 15 patients had focal to bilateral tonic-clonic seizures, 10 patients had focal unaware seizures, 3 patients had focal aware seizures, 1 patient had a generalized tonic-clonic seizure, and 1 patient had epileptic spasms. The epileptogenic lesions were lateralized to the right hemisphere in 12 patients and to the left hemisphere in 15 patients. In addition, 1 patient had bilateral lesions, and 2 patients had a normal MRI. In terms of localization, 18 patients had a temporal lesion, 6 patients had extratemporal lesions, 4 patients had both temporal and extratemporal lesions, and 2 patients had a normal MRI. The mean duration of epilepsy prior to surgery was 59.5 months. At the time of surgery, the mean age was 13.7 years. Following surgery, the mean follow-up duration was 45.9 months (Table I).

The most commonly observed pathologies were tumors, MTS, and FCD. Detailed MRI characteristics are presented in Table II. Of the 30 patients, 9 underwent lesionectomy alone: 6 had extratemporal lesions, 2 had temporal lesions, and 1 patient had both temporal and extratemporal lesions. A total of 19 patients underwent lesionectomy with anterior temporal lobectomy (ATL), 1 patient underwent selective amygdalohippocampectomy and another patient underwent a complete right hemispherectomy. The surgical approach varied depending on the location of the lesion, but the goal in all cases was to achieve complete or maximal resection of the EZ (Table III). Histopathological analysis confirmed the presence of tumors in 16 patients, accounting for 53.3% of those examined. The most common tumor types identified were gangliogliomas (7 patients), dysembryoplastic neuroepithelial tumors (4 patients) and astrocytomas (3 patients). In addition, 3 patients with tuberous sclerosis complex had cortical tubers. FCD was identified in 9 patients, representing 30% of the cases, with 5 of them also exhibiting MTS. Gliosis was present in 5 cases, either as an isolated finding or in combination with other pathologies (Table SI).

**Figure 1:**
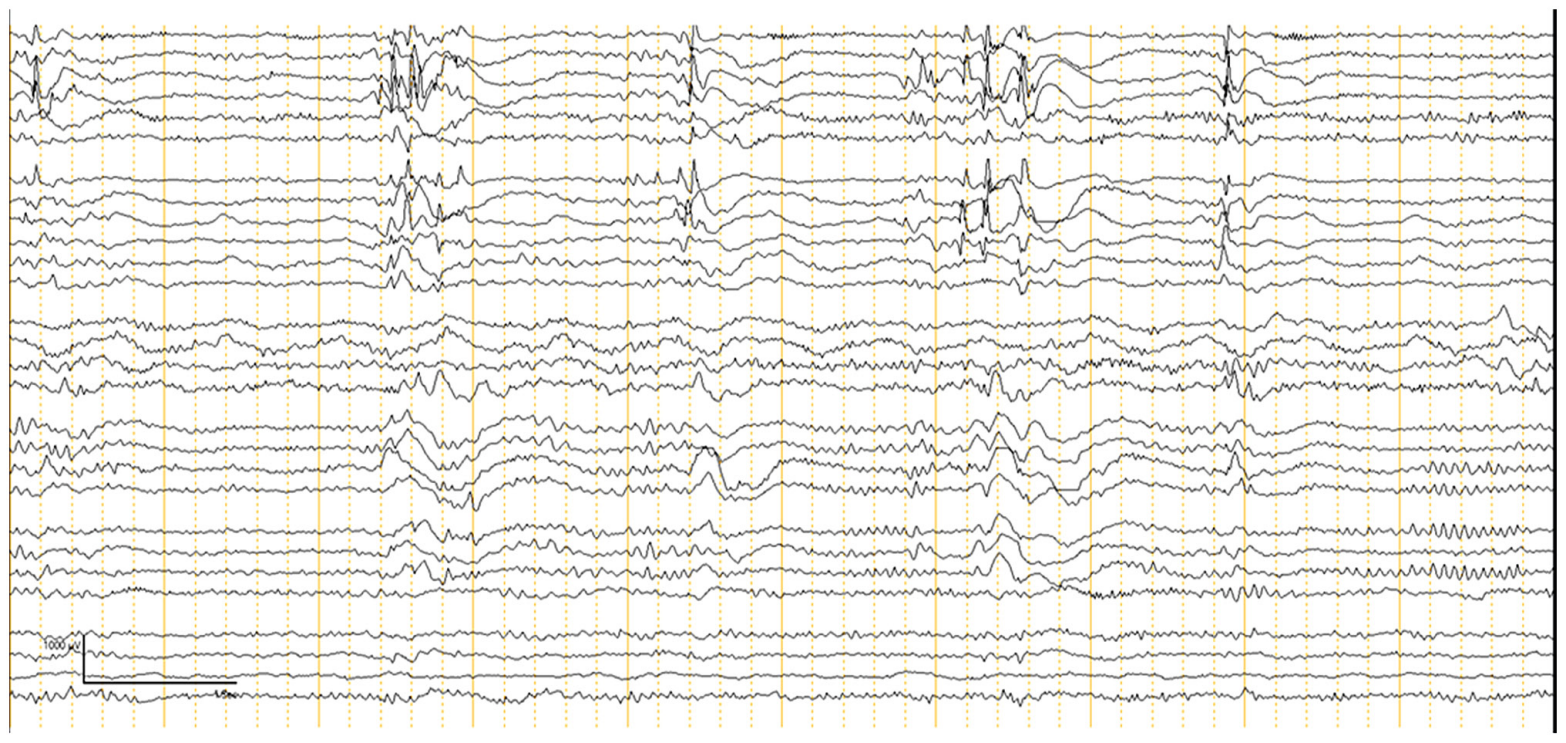
Pre-resection electrocorticography recording. This recording captures baseline cortical activity before resection, showing periodic epileptiform discharges over the inferior frontal region and some epileptiform discharges over the mesial and lateral frontal regions, assisting in identifying epileptogenic zones.

**Figure 2:**
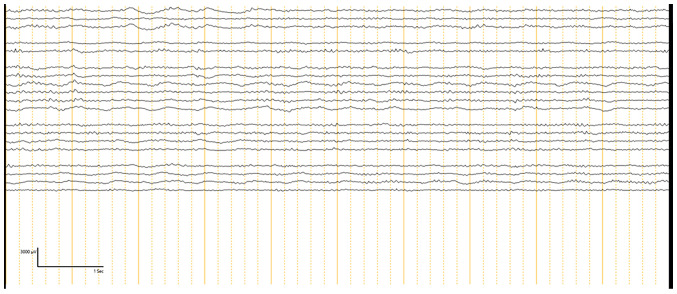
Post-resection electrocorticography recording. This recording captures brain activity after the resection of targeted areas. This recording is used to assess the immediate impact of the surgery and verify that the epileptogenic tissue has been removed.

ECoG was performed on all 30 patients to guide the extent of epileptogenic tissue resection. The ECoG findings were categorized into two phases: Pre-resection (Fig. 1) and post-resection (Fig. 2). Pre-resection spikes were noted in all 30 cases, indicating the presence of epileptogenic foci. The most prominent spikes were found in areas identified by MRI as locations of seizure focus localization. ECoG confirmed some cases where more extensive resections were necessary due to the inability of the previous MRI to show the full extent of the affected area. Following lesion resection, post-resection ECoG was conducted to determine if any epileptogenic activity remained. Out of the 30 patients, 13 patients (43.3%) had no residual IEDs. These patients were most likely to achieve seizure freedom post-operatively. However, 17 patients (56.6%) showed attenuated spikes on post-resection ECoG. In these cases, further resection was not possible, either due to the proximity of the epileptogenic tissue to eloquent brain areas or because the decision was made to minimize the risk of neurological deficits. Among these patients, 5 achieved Engel's Class II or III seizure outcomes, while 3 patients had Engel's Class IV outcomes, indicating persistent seizure activity post-operatively (Tables IV and V). Details of patients who were seizure-free (Engel Class I) are presented in Table IV, while details of patients who had Engel Class II-IV outcomes are presented in Table V. Of the patients that were seizure-free, 5 patients were completely off anti-seizure medications (ASMs), while 14 patients had their ASMs either tapered or maintained at the same dose if 2 years of post-operative seizure freedom had not yet passed (Table IV). Patients who did not achieve seizure freedom continued on ASMs with some adjustments.

Post-operative MRIs confirmed complete excision in 14 patients, with 7 patients showing residual lesions. Post-operative MRIs were not performed for 9 patients. Of the patients with residual lesions, 4 were classified as Engel's Class III or IV (Tables IV and V). Post-operative EEG was normal in 18 patients (60%), while EEG abnormalities were associated with poorer outcomes. In addition, 4 patients experienced transient contralateral hemiparesis affecting gross motor function, attributed to a lesion around the motor cortex, which resolved in 3 to 4 weeks. No other neurological deficits were observed in the remaining patients, indicating overall favorable neurological outcomes in this cohort.

## Discussion

The present study examined the role of ECoG in guiding epilepsy surgery and predicting seizure outcomes in a cohort of patients with DRE. The findings demonstrated the utility of ECoG in aiding resection decisions, especially in achieving seizure freedom for the majority of patients, with 63.3% achieving Engel Class I outcomes, indicating complete seizure freedom.

The results are consistent with previous studies regarding the role of ECoG in identifying EZs and guiding resection to achieve seizure freedom. The absence of IEDs on post-resection ECoG was associated with Engel Class I outcomes in 13 patients (43.3%), aligning with findings from Ravat *et al* (12) and Greiner *et al* (13), which highlight the predictive value of the absence of IEDs for positive postoperative results. ECoG enabled adjustments to the extent of resection during surgery, helping to reduce seizure recurrence, particularly for patients with temporal lesions, which were the most common pathology in the present cohort.

The high incidence of temporal lobe pathologies, such as MTS and FCD, highlights the surgical challenge of complete resection, particularly when lesions are near eloquent areas. In these instances, a decrease rather than total absence of post-resection ECoG IEDs often indicates worse outcomes (Engel Classes II-IV). This is in line with findings from studies by Sugano *et al* (14) and Fernandez and Loddenkemper (15), which showed that persistent spikes in ECoG were associated with lower rates of seizure freedom. The pre-resection ECoG identified epileptogenic activity in all patients, offering valuable insights into delineating the boundaries of the EZ, particularly when MRI results are inconclusive (16,17). Among patients who did not achieve complete absence of IEDs on ECoG, 56.6% exhibited reduced spikes after resection. This subset had lower rates of seizure freedom, with some falling into Engel Class II-IV categories. These findings emphasize the significance of striving for maximal resection while ensuring safety, as residual epileptogenic activity tends to correlate with less favorable seizure outcomes.

According to the present findings, the majority of lesions were located in the temporal lobe, with MTS, FCD, and tumors being the most common pathologies. These types of lesions often require extensive resection, especially in cases of MTS or FCD where the EZ may extend beyond the visible lesion. Consistent with other studies, patients with tumors or FCD were more likely to have epileptogenic activity beyond the lesion itself, highlighting the importance of performing ECoG to determine the extent of resection (12,18,19). In the subgroup of patients who underwent ATL in addition to lesionectomy, seizure freedom was achieved more frequently than in patients undergoing lesionectomy alone, demonstrating the effectiveness of combined surgical methods.

Post-operative MRI findings in the present study underscored the critical role of imaging in evaluating surgical success and its impact on seizure outcomes. Complete resection of epileptogenic lesions, achieved in 14 patients (46%), was strongly linked to favorable outcomes, with all patients attaining Engel Class I or II status indicating significant seizure control or freedom. These results emphasize the importance of MRI in confirming lesion removal and establishing a foundation for ongoing care and monitoring. By contrast, residual lesions were identified in 7 patients, of whom 4 experienced persistent or recurrent seizures (Engel Class III or IV). This highlights the imperative of maximizing lesion resection to optimize seizure control, while carefully managing the risks of neurological deficits, particularly in cases involving lesions near eloquent brain areas.

The post-operative EEG findings further reinforced the significance of thorough resection. Among the patients, 18 (60%) exhibited normal EEG patterns post-surgery, strongly associated with improved seizure outcomes. However, persistent IEDs were observed in 4 patients, aligning with poorer seizure control. These findings are consistent with prior research highlighting the predictive value of EEG in postoperative evaluations (20,21). By detecting residual epileptogenic activity, EEG serves as a crucial tool for identifying patients at risk of seizure recurrence and guiding the need for additional therapeutic strategies. Together, post-operative MRI and EEG offer a complementary approach, providing a comprehensive framework to predict and enhance surgical outcomes in epilepsy care.

Additional research is needed to explore advanced ECoG techniques, such as high-resolution grid electrodes to improve the accuracy of localizing EZs. Combining ECoG with novel imaging tools such as PET or functional MRI in future studies could enhance the precision of EZ localization, particularly in cases of extratemporal epilepsy. Establishing a stronger association between ECoG results and histopathological findings could guide tailored surgical strategies based on specific lesion types. The present study provides evidence supporting the effectiveness of intraoperative ECoG in improving surgical outcomes for patients with DRE. The observed seizure freedom rates align with those reported in high-income countries, highlighting the feasibility and value of ECoG-guided resections even at local healthcare systems.

This research addresses a significant gap in the literature by contributing long-term outcome data from the EMR (Saudi Arabia), where epilepsy remains highly prevalent, yet access to surgical care is often restricted. Unlike Western centers with established surgical pathways and monitoring protocols, institutions in our region commonly face challenges such as limited specialist availability, low public awareness, and delayed referrals (22-24).

The retrospective design of the present study introduces limitations, including potential recall and documentation bias, as data were collected from existing medical records rather than prospectively. The single-center setting and small sample size may also limit the external validity and generalizability of the findings to broader populations. Post-operative MRI was not obtained in almost a third of the patients due to logistical constraints. This missing data may introduce selection bias and limit the strength of conclusions drawn regarding the association between the extent of resection and seizure outcomes. Future research should aim for prospective, multicenter studies with standardized follow-up protocols and multidimensional outcome assessments to enhance the robustness and applicability of findings.

## Supplementary Material

Histological details of patients.

## Figures and Tables

**Table I:** Demographic and clinical characteristics of patients.

Categories	No. of patients (N=30)	Percentage (%) of patients
Sex		
Male	16	53.3
Female	14	46.6
Handedness		
Right	26	86.6
Left	4	13.3
CNS examination		
Normal	28	93.3
Right hemiparesis	2	6.6
Seizure type		
Focal to bilateral tonic-clonic	15	50
Focal unaware	10	33.3
Focal aware	3	10
Generalized tonic-clonic	1	3.3
Epileptic spasms	1	3.3
Lesion lateralization on MRI		
Right	12	40
Left	15	50
Bilateral	1	3.3
Normal	2	6.6
Lesion localization on MRI		
Temporal	18	60
Extratemporal	6	20
Both	4	13.3
Normal MRI	2	6.6
Mean (range) epilepsy duration before surgery (months)	59.5 (3-204)	
Mean (range) age at surgery (years)	13.7 (3-29)	
Mean (range) follow-up duration (months)	45.9 (7-120)	

CNS, central nervous system; MRI, magnetic resonance imaging.

**Table II:** MRI characteristics of patients.

Lesion location	Lesion type	No. of patients
Temporal	MTS + FCD	4
	MTS + tumor	1
	MTS + gliosis	1
	Tumor	8
	Tumor + FCD	4
	FCD	2
	Gliosis	2
Extra-temporal	FCD	2
	Tumors	3
	Gliosis + calcification	1
Both temporal and	Gliosis	1
extra-temporal	FCD	1

MTS, mesial temporal sclerosis; FCD, focal cortical dysplasia.

**Table III:** Details of surgery.

Type of surgery	Details	No. of patients
Lesionectomy alone		9
	Temporal	2
	Extra-temporal	6
	Both	1
Lesionectomy plus		21
	Anterior temporal lobectomy	19
	Selective amygdalo-hippocampectomy	1
	Right hemispherectomy	1

**Table IV:** Details of patients who achieved seizure freedom (Engel Class I).

Serial no.	MRI	Post-resection IEDs on iECoG	Histopathology	Type of surgery	Post-op EEG	Post-op MRI
1	LT occipital-parietal old infarction	Attenuated	Gliosis	Lesionectomy alone	Abnormal	Not performed
2	RT frontal FCD	Absent	FCD IIB	Lesionectomy alone	Not performed	Not performed
3	LT temporal lesion	Attenuated	Ganglioglioma	Lesionectomy + ATL	Normal	Complete excision
4	LT temporal lesion + MTS	Absent	Ganglioglioma + MTS	Lesionectomy + ATL	Normal	Complete excision
5	Multiple cortical tubers (+ RT temporal)	Absent	Tuber + FCD IIIB	Lesionectomy + ATL	Normal	Not performed
6	LT hippocampal atrophy	Absent	MTS + FCD IIIa	Lesionectomy + ATL	Abnormal	Not performed
7	LT temporal lesion	Absent	Ganglioglioma + FCD	Lesionectomy + ATL	Not performed	Complete excision
8	LT temporal lesion	Attenuated	Astrocytoma + FCD IIIB	Lesionectomy + ATL	Normal	Complete excision
9	RT temporal lesion	Attenuated	Ganglioglioma + FCD	Lesionectomy + ATL	Normal	Complete excision
10	RT temporal lesion	Attenuated	DNET	Lesionectomy alone	Normal	Residual lesion
11	LT temporal lesion (incomplete previous resection)	Absent	Ganglioglioma	Lesionectomy + ATL	Not performed	Complete excision
12	LT temporal lesion	Attenuated	Ganglioglioma	Lesionectomy + ATL	Normal	Complete excision
13	RT temporal lesion	Absent	Ganglioglioma + FCD IIIB	Lesionectomy + ATL	Normal	Not performed
14	Unremarkable	Attenuated	FCD	Lesionectomy + ATL	Normal	N/A
15	LT parietal lesion	Absent	DNET	Lesionectomy alone	Normal	Complete excision
16	RT temporal MTS	Attenuated	FCD Ia	Lesionectomy + ATL	Normal	Complete excision
17	LT temporal lesion	Absent	Astrocytoma	Lesionectomy alone	Normal	Residual lesion
18	RT temporal lesion	Attenuated	Pleomorphic xanthroastrocytoma	Lesionectomy + ATL	Normal	Complete excision
19	LT temporal lesion	Absent	MTS + FCD IIIa	Lesionectomy + ATL	Abnormal	Complete excision

MRI, magnetic resonance imaging; IEDs, interictal discharges; iECoG, intraoperative electrocorticography; EEG, electroencephalography; LT, left; RT, right; FCD, focal cortical dysplasia; ATL, anterior temporal lobectomy; MTS, mesial temporal sclerosis; DNET, dysembryoplastic neuroepithelial tumor; N/A, not available.

**Table V:** Details of patients not achieving seizure freedom.

No.	MRI	Post-resection IEDs ECoG	Histopathology	Type of surgery	Post-op EEG	Post-op MRI	Engel Class
1	LT temporal FCD	Absent	MTS + FCD IIIa	Lesionectomy + ATL	Abnormal	Complete excision	Class II
2	RT frontal gyrus rectus FCD	Attenuated	MTS + FCD IIIa	Lesionectomy alone	Abnormal	Residual lesion	Class IV
3	LT temporal MTS	Attenuated	MTS + FCD IIIa	Lesionectomy + ATL	Abnormal	Not performed	Class III
4	RT inferior frontal lesion	Absent	DNET	Lesionectomy alone	Normal	Complete excision	Class II
5	RT TPO post-ischemic changes	Attenuated	Gliosis	RT hemispherotomy	Abnormal	Residual lesion	Class II
6	RT LV SEGA + multiple tubers	Attenuated	Tubers	Lesionectomy + ATL	Normal	Not performed	Class II
7	Unremarkable	Absent	MTS + gliosis	Lesionectomy + SAH	Normal	Complete excision	Class II
8	LT temporal atrophy	Attenuated	Gliosis	Lesionectomy + ATL	Abnormal	Residual lesion	Class IV
9	RT temporal occipital lesion	Attenuated	FCD	Lesionectomy alone	Abnormal	Residual lesion	Class IV
10	RT temporal lesion	Attenuated	Gliosis	Lesionectomy + ATL	Normal	Not performed	Class III
11	LT cingulate gyrus lesion	Attenuated	DNET	Lesionectomy alone	Normal	Residual lesion	Class III

MRI, magnetic resonance imaging; IEDs, interictal discharges; iECoG, intraoperative electrocorticography; EEG, electroencephalography; LT, left; FCD, focal cortical dysplasia; ATL, anterior temporal lobectomy; RT, right; MTS, mesial temporal sclerosis; TPO, temporo-parietal and occipital; LV, lateral ventricle; SEGA, subependymal giant cell astrocytoma; SAH, selective amygdalohippocampectomy.

## Data Availability

The data generated in the present study are included in the figures and/or tables of this article.
