# Supplementary material for: Intraoperative electrocorticography in focal drug-resistant epilepsy: A 10-year retrospective single-center study
Source: Biomed Rep. 2025 Sep 1;23(5):171. doi: 10.3892/br.2025.2049 (PMC12415504; doi:10.3892/br.2025.2049)
Supplement: Histological details of patients. [file Supplementary_Data.pdf]

Table SI. Histological details of patients.

Histology	No. of patients
Tumors	16
GG	7
GG alone	3
GG + FCD	3
GG + MTS	1
DNET	4
Astrocytoma	3
Astrocytoma alone	2
Astrocytoma + FCD	1
Tubers	2
Tubers alone	1
Tubers + FCD	1
FCD alone	4
FCD + MTS	5
Gliosis alone	4
Gliosis + MTS	1

GG, ganglioglioma; DNET, dysembryoplastic neuroepithelial tumor; FCD, focal cortical dysplasia; MTS, mesial temporal sclerosis.
